# Robust Vessel Segmentation in Fundus Images

**DOI:** 10.1155/2013/154860

**Published:** 2013-12-12

**Authors:** A. Budai, R. Bock, A. Maier, J. Hornegger, G. Michelson

**Affiliations:** ^1^Pattern Recognition Lab, Friedrich-Alexander University, Erlangen-Nuremberg, 91058 Erlangen, Germany; ^2^International Max Planck Research School for Optics and Imaging (IMPRS), 91058 Erlangen, Germany; ^3^Erlangen Graduate School in Advanced Optical Technologies (SAOT), 91052 Erlangen, Germany; ^4^Department of Ophthalmology, Friedrich-Alexander University, Erlangen-Nuremberg, 91058 Erlangen, Germany; ^5^Interdisciplinary Center of Ophthalmic Preventive Medicine and Imaging (IZPI), 91054 Erlangen, Germany

## Abstract

One of the most common modalities to examine the human eye is the eye-fundus photograph. The evaluation of fundus photographs is carried out by medical experts during time-consuming visual inspection. Our aim is to accelerate this process using computer aided diagnosis. As a first step, it is necessary to segment structures in the images for tissue differentiation. As the eye is the only organ, where the vasculature can be imaged in an in vivo and noninterventional way without using expensive scanners, the vessel tree is one of the most interesting and important structures to analyze. The quality and resolution of fundus images are rapidly increasing. Thus, segmentation methods need to be adapted to the new challenges of high resolutions. In this paper, we present a method to reduce calculation time, achieve high accuracy, and increase sensitivity compared to the original *Frangi* method. This method contains approaches to avoid potential problems like specular reflexes of thick vessels. The proposed method is evaluated using the *STARE* and *DRIVE* databases and we propose a new high resolution fundus database to compare it to the state-of-the-art algorithms. The results show an average accuracy above 94% and low computational needs. This outperforms state-of-the-art methods.

## 1. Introduction

**Figure 1:**
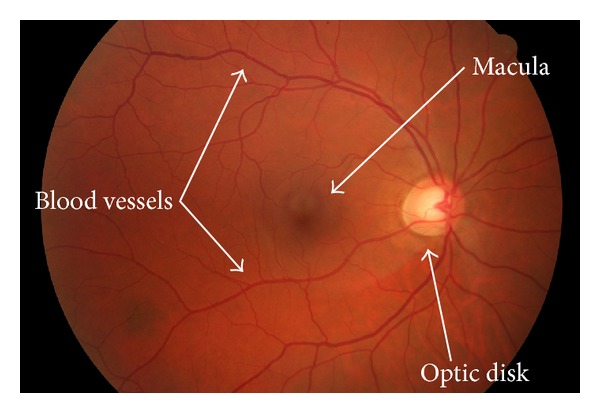
An example of eye-fundus image: the macula is shown in the middle, the optic disk is to the right, and the blood vessels are entering and leaving the eye through the optic disk.

In ophthalmology the most common way to examine the human eye is to take an eye-fundus photograph and to analyse it. During this kind of eye examinations a medical expert acquires a photo of the eye-background through the pupil with a fundus camera. The analysis of these images is commonly done by visual inspection. This process can require hours in front of a computer screen, in particular in case of medical screening. An example fundus image is shown in Figure 1.

Our goal is to speed up the diagnosis by processing the images using computer algorithms to find and highlight the most important details. In addition we aim to automatically identify abnormalities and diseases with minimal human interaction. Due to the rapidly increasing spatial resolution of fundus images, the common image processing methods which were developed and tested using low resolution images have shown drawbacks in clinical use. For this purpose, a new generation of methods needs to be developed. These methods need to be able to operate on high resolution images with low computational complexity. In this paper, we would like to introduce a novel vessel segmentation method with low computational needs and a public available high resolution fundus database with manually generated gold standards for evaluation of retinal structure segmentation methods. The proposed algorithms include modifications to the method proposed by Frangi et al. [1] to decrease the running time and to segment specular reflexes of thick vessels, which are not visible in lower resolution fundus images.

The structure of the paper is as follows. We describe the proposed methods in detail in Section 3. In Section 4, we present the evaluation methods and databases, including our proposed high resolution fundus database, while Section 5 presents the quantitative results. In Sections 6 and 7, the computational complexity and robustness of the proposed algorithm are analyzed. This is followed by a Discussion in Section 8 and the Conclusions in Section 9.

## 2. Related Work

Retinal vessel segmentation is a challenging task and has been in the focus of researches all over the world for years. During this time many different algorithms were published [2]. The segmentation algorithms can be classified into two main groups: in unsupervised and supervised methods. Unsupervised methods classify vessels using heuristics, while supervised methods learn a criteria system automatically using prelabeled data as gold standard. We focus on heuristic methods, as supervised methods need a large training set for each camera setup. Heuristic methods instead require a set of parameters, which need to be adapted to the camera setup. Thus, they are much more independent from the test dataset during their development. A more detailed review of the segmentation and other retinal image processing algorithms can be found in the articles published by Kirbas and Quek [2] and Patton et al. [3].

Early, but one of the most common approaches for fundus images are the matched-filter approaches. One of the first methods was presented by Chaudhuri et al. [4]. It fits predefined vessel profiles with different sizes and orientations to the image to enhance vessels. Similar methods and improvements were published later on by different authors [5–8]. Early implementations of these methods were using a simple thresholding step to obtain a vessel segmentation. Sometimes these methods were combined with other approaches [9–12]. For example, Zhang et al. [12] combined matched filters with a method based on the Hessian matrix [1]. The matched filters provide high quality results, but the main disadvantage of these methods is their requirement for vessel profiles and comparisons of large regions for each pixel in the image, resulting in long computational time. The quality of the segmentation results heavily depends on the quality and size of the used vessel profile database. This can be specific towards ethnicity, camera setup, or even eye or vascular diseases, which reduces its applicability.

Some of the algorithms are specialized to segment only one or more objects, which are marked by a user or in a preprocessing step. These methods are usually not analyzing the whole image but the neighborhood of the already segmented regions. Region growing [13, 14] and tracking algorithms [15–18] are good examples for such kind of segmentation methods. The region-growing approaches are trying to increase the segmented area with nearby pixels based on similarities and other criteria. These methods are one of the fastest approaches, while they may have problems at specific regions of the image, where the vessels have lower contrast compared to the nearby tissues, for example, vessel endings or thin vessels. In this case the region growing can segment large unwanted areas. Vessel tracking algorithms are more robust in those situations. They try to find a vessel-like structure in the already segmented region and track the given vessels. These algorithms can recognize vessel endings much easier, but they may have difficulties at bifurcations and vessel crossings, where the local structures do not look like usual vessels anymore. Hunter et al. [17] published a postprocessing step to solve some of these situations.

Other common segmentation approaches are model-based methods. The most known and commonly used ones are active contour-based methods, level-sets, and the so-called snakes [19]. The early snake-based algorithms start with an initial rough contour of the object, which is iteratively refined driven by multiple forces. In an optimal case, the forces reach their equilibrium exactly on the object boundaries. These methods are sensitive to their parameterization, while they may have problems if they have to segment thick and thin vessels in the same time. Thus, the parameters have to be set and refined manually by the user. The snakes in this form are mostly used in MR [20] or X-ray angiographic images [21] to segment pathologies and organs. The snake-based retinal vessel segmentation methods usually apply a vessel tracking framework to find the edges or the centerline of the vessels and track them using snakes [17, 22, 23]. This way the snakes are used to track only a vessel edge and the algorithm has less problems with vessel endings and different vessel thicknesses. Thus, their parameters are easier to optimize, but they inherit the problems of tracking algorithms with bifurcations and crossings.

Level-set methods provide a more robust solution than snakes. They are usually used in combination with other vessel enhancement techniques incorporating a smoothness constraint in their level set functions [24, 25].

For an automated segmentation method used in screening, the most important properties are robustness, efficiency, and the calculation time, because hundreds or thousands of images have to be processed each day. The state-of-the-art vessel segmentation methods [12, 22] usually have high computational needs and achieve an accuracy of 90% to 94% on eye-fundus images, with sensitivity of 60% to 70% and specificity above 99% on average [26]. This is due to the fact that approximately 85% of an image shows background structures. The high computational needs are due to multiple analysis of large regions to detect thick vessels. Thus, the computational needs of an algorithm is increasing exponentially with the diameter of the expected thickest vessel and the image resolution.

We present an algorithm based on the vessel enhancement method published by Frangi et al. [1] in combination with a multiresolution framework to decrease the computational needs and to increase the sensitivity by using a hysteresis thresholding. The method published by Frangi et al. [1] is a mathematical model-based approach and extracts vesselness features based on measurements of the eigenvalues of the Hessian matrix. The Hessian matrix contains the second-order derivatives in a local neighborhood. The method assumes that the vessels are tubular objects; thus, the ratio of the highest and lowest eigenvalue should be high, while this ratio is close to one in regions of constant values. The method was developed for CT angiography images, but it is applied in a wide variety of vessel segmentation algorithms and detection of tubular objects in different modalities [1, 27]. One of the disadvantages is the computational requirement. As Frangi et al. [1] proposed, the method calculates the Hessian matrix and the given measures for increasing neighborhood sizes, until the neighborhood is bigger than the expected thickest vessel. Given high resolution images, this can easily increase to 20 to 30 iterations per pixel.

## 3. Methods

All methods that were used to analyze the images are described in this section. First, we introduce the method proposed by Frangi et al. [1], which provides the basis of this work. This is followed by the description of the proposed method: the preprocessing steps in Section 3.2.1 and the used resolution hierarchy in Section 3.2.2. After that the vessel enhancement method is described to highlight the main differences to the Frangi method. We have chosen the method published by Frangi et al. [1] as a base for our own work, because it features some attractive properties.

- High accuracy is expected based on preliminary research [1, 27]. For further information please see Section 5.1. In comparison, our implementation of this method achieved a high accuracy.
- No user interaction is required, except for setting a few parameters.
- It is able to segment nonconnected objects without complex initialization steps. This is necessary in case of some abnormalities and in case of young patients, where reflections may disconnect vessels.

### 3.1. Frangi's Algorithm

To understand the proposed method, the reader should know the method by Frangi et al. [1]. Thus, in this section, we will introduce the method as it was published by Frangi et al. [1] in 1998.

The Hessian matrix of an *n* dimensional continuous function *f* contains the second-order derivatives. As we are working on a 2-dimensional image, our Hessian matrix is given as

(1) $$\begin{array}{c} H\left(f\right)=\left(\begin{array}{cc} \frac{\partial^{2}f}{\partial x^{2}} & \frac{\partial^{2}f}{\partial x\partial y}\\ \frac{\partial^{2}f}{\partial y\partial x} & \frac{\partial^{2}f}{\partial y^{2}} \end{array}\right). \end{array}$$

The Hessian matrix *H*_0,*s*_ is calculated at each pixel position *x*_0_ and scale *s*. Frangi used *s* as the standard deviation (*σ*) of Gaussians to approximate the second-order derivatives. A vesselness feature *V*_0_(*s*) is calculated at pixel position *x*_0_ from the eigenvalues *λ*_1_ < *λ*_2_ of the Hessian matrix *H*_0,*s*_ using equations of “dissimilarity measure” *R*_*B*_ and “second order structuredness” *S*

(2) $$\begin{array}{c} R_{B}=\frac{\lambda_{1}}{\lambda_{2}},\\ S=\sqrt{\lambda_{1}^{2}+\lambda_{2}^{2}},\\ V_{0}\left(s\right)=\{\begin{array}{cc} 0, & \text{if}\;\;\lambda_{2}>0,\\ \exp\left(-\frac{R_{B}^{2}}{2\beta^{2}}\right)\left(1-\exp\left(-\frac{S^{2}}{2c^{2}}\right)\right), & \end{array} \end{array}$$

where *β* and *c* are constants which control the sensitivity of the filter. *R*_*B*_ accounts for the deviation from blob-like structures, but can not differentiate background noise from real vessels. Since the background pixels have a small magnitude of derivatives and, thus; small eigenvalues, *S* helps to distinguish between noise and background.

The authors suggest to repeat the same calculations for varying sigma values from one to the thickest expected vessel thickness with an increment of 1.0 to enhance vessels with different thicknesses. The results are combined by a weighted maximum projection. In our implementation we added a thresholding step after the combination and optimized the parameters to reach the highest accuracy.

### 3.2. Proposed Method

After the preprocessing steps, we apply the same equations as described by Frangi et al. [1] for each resolution level with the same predefined sigma value.

Hence, we do not increase the sigma value linearly and apply the filter multiple times on the image as it was proposed by Frangi et al. [1]. In our case the sigma is always set to a small constant, while we apply the same method on copies of the input image with reduced resolutions. Thus, the parameter *s* of the original method corresponds to the resolution of the image, instead of the standard deviation of a Gaussian.

**Figure 2:**
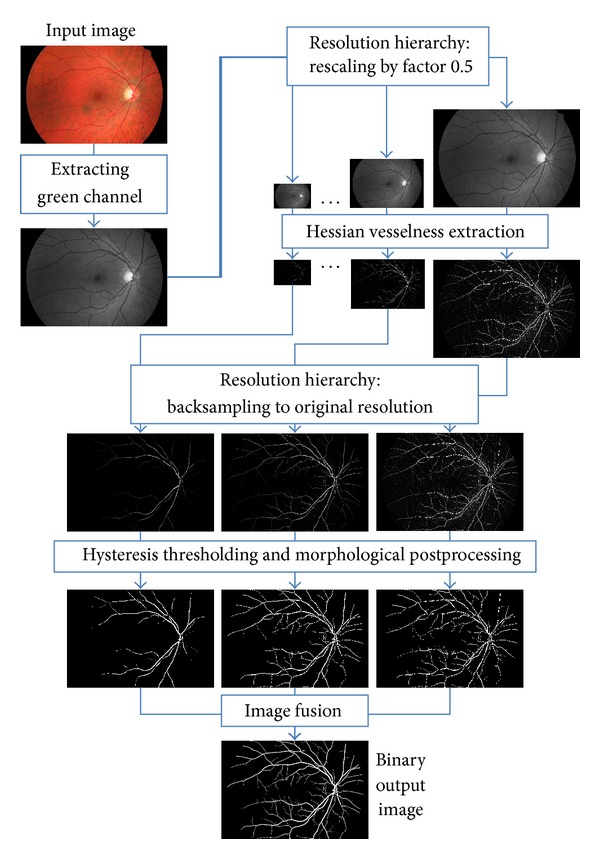
Pipeline of the proposed segmentation algorithm.

The proposed algorithm of our method is illustrated in Figure 2. Each of the steps will be discussed in detail in the next sections.

#### 3.2.1. Preprocessing

**Figure 3:**
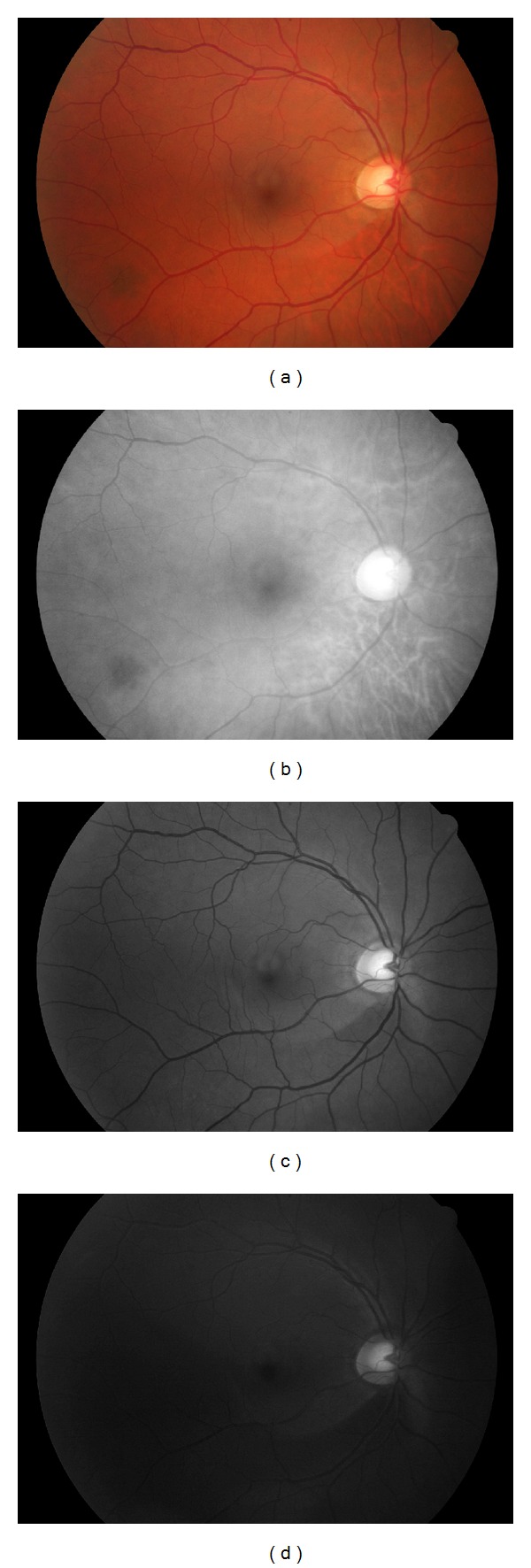
A fundus image (a) and its RGB decomposition showing the oversaturated red (b), the well-illuminated green (c), and the under-illuminated blue (d) channels.

The input images are digital color fundus photographs like the one in Figure 1. During the analysis we restrict ourselves to the green channel. It has the highest contrast between the vessels and the background, while it is not underilluminated or oversaturated like the other two channels, see Figure 3 for an example. Histogram stretching [28] and bilateral filtering [29] are applied to the green channel. The histogram stretching increases the contrast to make it easier for the algorithm to detect small changes and distinguish different tissues. The bilateral filtering [30] is a special denoising algorithm, which smooths intensity changes, while preserving the boundaries of different regions or tissues. This step reduces false positive detections caused by the texture of the background. After these modifications of the data, we can apply our resolution hierarchy described in the next section.

#### 3.2.2. Resolution Hierarchy

**Figure 4:**
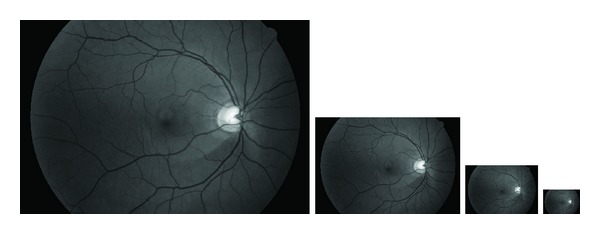
Example of a Gaussian resolution hierarchy using only the green channel of the input image and its three reduced resolution versions.

In a resolution hierarchy copies of the input image with reduced resolutions are generated; see Figure 4. By doing so, we calculate the Hessian matrix always for a small neighborhood which decreases the computational needs. The reduction is done by a subsampling followed by a low pass filtering to lower high jumps in intensities. The highest resolution level of the resolution hierarchy contains the original image, and all additional levels contain the image with a halved width and height compared to the previous level. For low resolution images, where the vessel thickness is not more than 5 to 10 pixels, 2 to 3 levels are sufficient, while images with higher resolutions may require additional levels. Compared to more than 20 iterations for the Frangi method, this means a speedup of a factor of 10. The vessel enhancement of the Frangi algorithm is applied on each resolution level with a standard deviation *σ* = 1.0.

**Figure 5:**
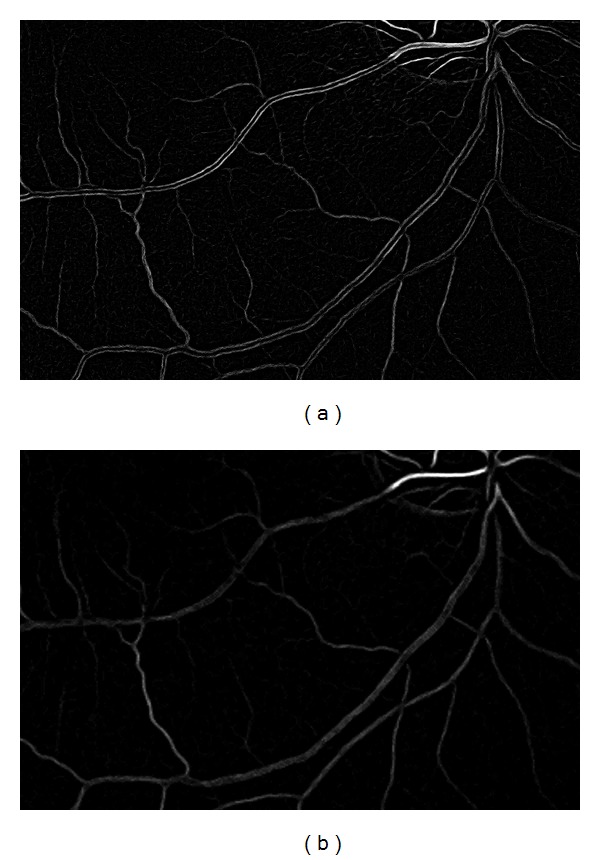
The resized vesselness images of two different resolution levels. In the highest resolution level (a) the enhanced image shows more details, while the result of a lower resolution level (b) shows a more accurate segmentation of thick vessels.

Sometimes the flash of the camera causes a shining centerline on thick vessels. An additional correction method was developed to remove these specular reflection artifacts in the reduced resolution levels. The resulting images of the vessel enhancement are resized again using bilinear interpolation to the same resolution as the input image.  Figure 5 shows the result of this resizing on two different resolution copies of the same region.  Figure 5(a) had a high resolution and the result shows finer details, but the thickest vessels are not enhanced correctly.  Figure 5(b) had a much lower resolution. Thus, the fine details disappeared, but the extraction of thick vessels were more accurate.

#### 3.2.3. Specular Reflex Correction

As mentioned before, the flash of the camera may cause a bright specular reflex in the middle of thick vessels. Because of these reflections, the Hessian-based filter will have a much lower response. In our algorithm we developed a filter to be used on the highest level of our resolution pyramid to reduce the effect of these reflections. In this level only thick vessels are detected. We consider a 3 × 3 neighborhood for each pixel. If the center pixel has a lower value than two neighboring pixels in opposite directions in the vessel enhanced image, but higher value than the same two pixels in the fundus image of the same resolution level, then the center pixels are affected by specular reflex. In this case the two neighboring pixel's value will be interpolated to update the center pixel's value.

#### 3.2.4. Hysteresis Threshold

After the vessel enhancement is completed in each resolution level and the results are resized to original resolution, all of them are binarized by a thresholding algorithm proposed by Canny [31]. The method performs better than a single thresholding in cases where the intensity of the objects is at some places high, but in certain positions the contrast between object and background falls under noise level. In our case this object is the vessel tree, where thin vessels and boundary pixel intensities can have extreme low intensity values. This method uses two thresholding values instead of one to binarize a gray-scale image. Both threshold values have different roles in the thresholding process.

1. The first threshold is used to determine pixels with high intensities. It is required that this threshold is chosen in such a way that no background pixel can reach that value. Thus, we can label all the pixels above the threshold as “vessel pixels.”
2. We label all pixels below the second threshold value as “background pixel” and all pixels in between the two thresholds are considered “potential vessel pixels.” These potential vessel pixels are labeled as vessels only if they are connected to a pixel labeled “vessel pixel” through other potential vessel pixels.

The thresholding values are computed for each image that a given percent of the pixels is segmented as “vessel pixels.” Thus, the binarization is more robust to noise and intensity changes between images. They have to be optimized for each different protocol and field of view, where the ratio of vessel and background pixels is different in the resulting image. The binarization is used on each image separately.

#### 3.2.5. Postprocessing

The final segmented image is generated by applying a pixel-wise OR operator on the binarized images originated from the different resolution levels. This way if a vessel was detected in one of the images, then it will be visible in the combined binary image.

Afterwards a thinning function erodes the segmented region until it reaches the highest local gradient in the input image. This method avoids the slight oversegmentation in case that a thin vessel is detected in a higher level of the hierarchy.

**Figure 6:**
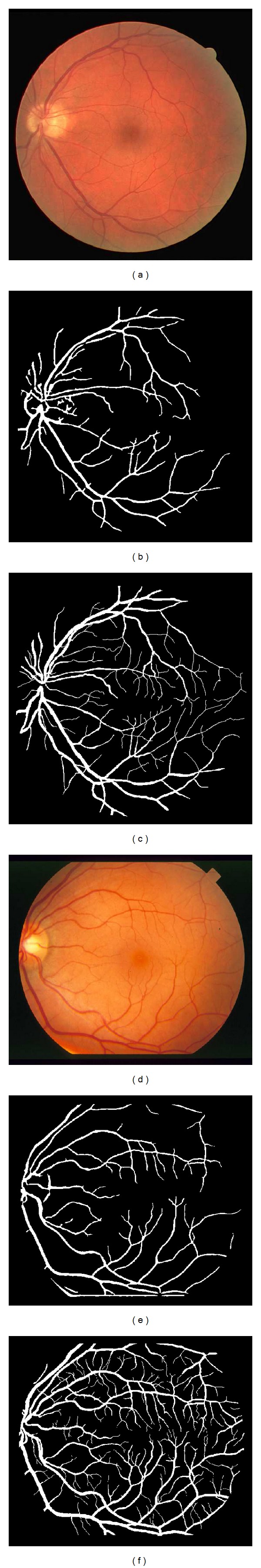
Example segmentation results on the DRIVE (upper row) and STARE (bottom row) public databases. From left to right: input fundus images, segmentation results, and gold standard images.

**Figure 7:**
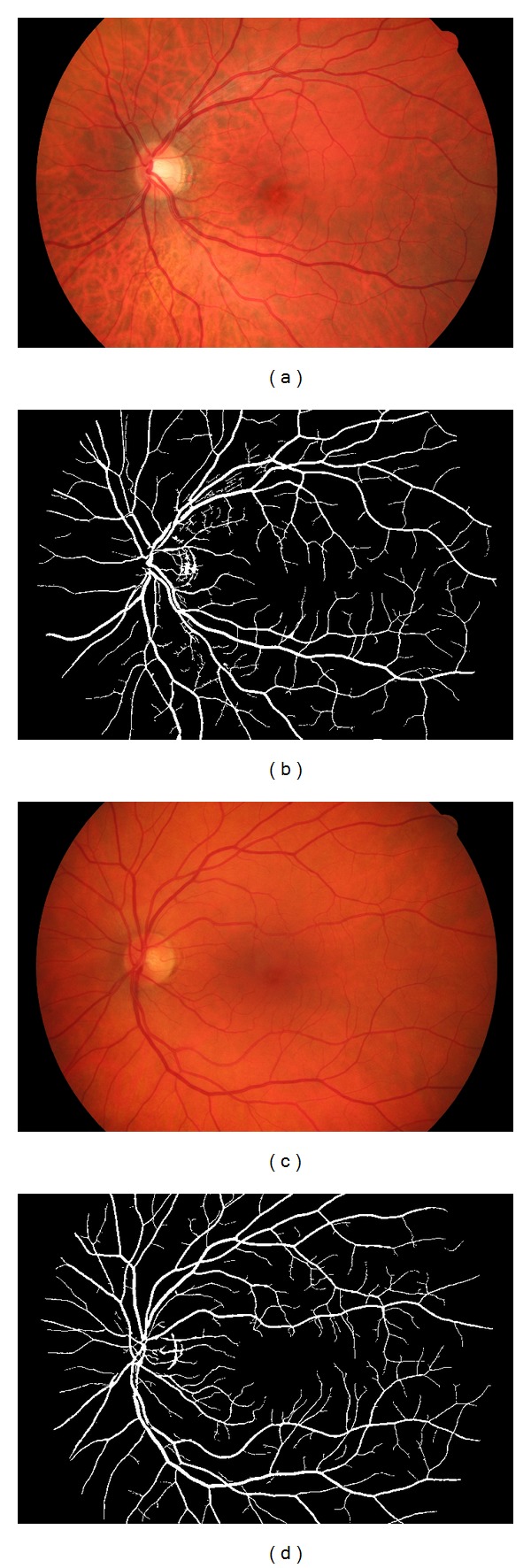
Example segmentation results on high resolution images with different illumination and background structures.

As a last step a small kernel (3 × 3) morphological closing operator is used to smooth the boundaries and object size analysis algorithms are applied to fill small holes in the vessel tree and remove small undesired objects. Some example of input images and the calculated segmentations are presented in Figures 6 and 7.

## 4. Evaluation

We applied the original Frangi vesselness extraction and our proposed framework on the commonly used DRIVE [26] and STARE [32] databases and on our high resolution public database [33] to compare our framework to the state-of-the-art methods and to evaluate their effectivity. These databases contain manual segmentations of experts as gold standard. Based on these gold standards we calculated the sensitivity (Se), specificity (Sp), and accuracy (Acc) of each method. Both already existing databases contain an additional manual segmentation and the DRIVE database contains some measurements of multiple algorithms.

We compare the computation time of the proposed algorithm and an implemented Frangi vesselness algorithm as proposed by Frangi et al. [1]. The two public databases were used to evaluate the efficiency and for comparison to other state-of-the-art algorithms. These two databases suffer from containing only low resolution images, while the proposed method was developed for high resolution images. Thus, the benefit of the resolution hierarchy is only slightly noticeable. Since high resolution images are becoming more common in clinical use, we evaluated our methods on the high resolution (3504 × 2336 pixels) images available [33], which were already used to evaluate other methods [6, 7]. The database contains 15 images of each healthy, diabetic retinopathy (DR), and glaucomatous eyes. The results of this evaluation are discussed in Section 5.2.

The technical details of the used image data are shown in Table 1. For each method, we applied the same parameter optimization process using a small subset of each database to assure that differences are not due to parameter settings. This algorithm sets the parameters to reach the highest possible accuracy without aiming at high sensitivity. Since the parameter is done using a small subset of the images, the results can be improved using a larger training set. Optimization based on a small subset may result in suboptimal settings for the whole dataset, but it shows the generalization capabilities of the method.

For the evaluation of computation times we always used the same common notebook equipped with a 2.3 GHz processor and 4 GB RAM and a single core implementation of the algorithms.

## 5. Results

### 5.1. Accuracy

The metrics calculated on the two public databases to analyze the effectivity of the algorithms are shown in Tables 2 and 3. During our development and in our comparisons we aimed at the highest possible accuracy. Therefore, we optimized the parameters of both—the proposed and the Frangi—methods. Thus, the parameters of the Frangi method and the proposed method are set to deliver the highest possible accuracy. This can result in a decreased sensitivity to gain specificity in order to increase the overall accuracy. This way the proposed method was able to reach the best accuracy using the DRIVE and high resolution fundus databases.

Both public databases contain a second manual segmentation made by a human observer, which was included in the comparison. We collected further results from published papers. For both databases the original method and the proposed method reached a high accuracy over 95% and 93%, respectively. As shown in Table 2, in case of the DRIVE database, this was enough to reach the highest accuracy. In case of the STARE database, as shown in Table 3, the sensitivity improved by 5% along with a slight increase in accuracy. Some examples of the segmentation results are shown in Figure 6.

The proposed algorithm and the original Frangi method were further tested on the three datasets of our own public high resolution fundus database [33].  Figure 7 shows two examples of input images and segmentation results of this database. As these images have much higher resolutions, we use more resolution levels in the hierarchy and higher *σ* values in the original Frangi algorithm. This enables detection of vessels with a higher diameter, but also increases the computation times. Tables 4 and 5 show the sensitivity, specificity, and accuracy of these methods using the high resolution fundus dataset. Each datasets with manually segmented gold standard images is available online [33] for other researchers to test and compare their algorithms.

### 5.2. Performance

Tested on the two public databases, the proposed method has a reduced calculation time by 18% in case of the STARE database and 16% in case of the DRIVE database, as shown in Table 6. The computation times were not available for most of the algorithms used for comparison in Section 5.1. Thus, these methods are excluded from the performance test. The resolution hierarchy made our proposed method faster on the low resolution images than the Frangi method. The speed improvement of the hierarchy is actually higher, but we used additional time for postprocessings and improvements, like filling the holes caused by central reflexes in the vessels and using a hysteresis thresholding in each resolution.

As the computation times of hysteresis threshold is rapidly increasing with the resolution, we tested the runtime using high resolution images to see if the gain using the resolution hierarchy is higher than the additional requirements of the thresholding.  Table 7 shows the computation times for these images.

The results show a calculation time difference of about 33.3%, which was less than 20% in case of low resolution images. This means that our proposed method performs the segmentation in higher resolution images faster in comparison to the original Frangi method.

## 6. Computational Complexity

To see the difference in computational complexity of both methods, we calculated the mathematical complexity of the Frangi method [1] and our proposed methods. As all segmentation methods need some pre- and postprocessing, we decided to calculate the mathematical complexity of the main vessel extraction only, plus our proposed direct modifications.

As a first step, we have to define the necessary parameters. Let *n* be the number of pixels in the input image, and define *t* as the highest expected vessel thickness which we would like to detect. With these two parameters, we can describe the complexity of the important components used in the algorithms:

- rescaling: *O*(*n*) for each image;
- calculating Hessian matrix: *O*(*t* ^2^) for each pixel;
- eigenvalue analysis: after calculating the Hessian matrix, it is independent of the parameters: *O*(*n*) for each image;
- postprocessing using mathematical morphology, and other operations: *O*(*n*) for each image;
- maximum image calculation: *O*(*m* · *n*) where *m* is the number of images;
- binarization by thresholding: *O*(*n*) for each image.

In case of the original method, calculation of the Hessian matrix is done *t* times for each pixel, with increasing *σ*. After that all the images are summarized and thresholded. These methods result in a complexity of *O*(*t* ^3^ × *n*): *t* × *n* pixels, and *O*(*t* ^2^) operations for each pixel, while the complexity of the other parts is neglectable.

The proposed method uses the rescaling. This results in a maximal pixel number of 1.5 × *n* to work on instead of *t* × *n*, and *t* is always set to one. Thus, the Hessian matrix calculation is done with a predefined *σ* = 1.0, which reduces the complexity to *O*(*n*). After rescaling to the original resolution, postprocessing and binarization are done in linear complexity. This gives a computational complexity of *O*(log⁡⁡(*t*)∗*n*): independently of the number of resolution levels, the maximal number of pixels is 1.5 × *n*, and *σ* is set to 1.0 which results in a computational complexity of *O*(*n*) before fusing the binarized images. With log⁡⁡(*t*) number of rescaled images, after the fusion, the complexity is *O*(log⁡⁡(*t*)∗*n*) with neglectable linear complexity of the postprocessing.

## 7. Robustness

To analyze the robustness and sensitivity of the method regarding changes in the parameters, we analyze it by further excluding some steps and changing the parameters.

As Table 8 shows, the algorithm is robust against changes in the parameters of pre- or postprocessing, except that not all of the processing steps are skipped. This increases the false positive values due to the appearance of small segmented noisy regions and also increases false negatives by not segmenting regions of vessels with specular reflexes.

The accuracy of the method improved surprisingly by increasing the *σ* to 2.0 for the vessel enhancement. Our analysis showed that the optimization using a small subset of images resulted in a suboptimal parameter set for the whole dataset. Changing the *sigma* value to 2.0 increased the sensitivity in multiple images, reaching an overall sensitivity over 0.7338 and accuracy over 0.9621.

## 8. Discussion

Our evaluation has shown that the proposed method not only has the highest accuracy using the high resolution images for which it was developed, but it has decent results using two lower resolution databases available online. This decrease is due to the slightly lower sensitivity caused by the lower image quality in the online databases. The proposed method has lower computational needs compared to the method proposed by Frangi et al. [1], as it was shown experimentally in Section 5.2 and mathematically proven in Section 6.

Furthermore, as shown in Section 7, the method is only slightly sensitive to the *σ* parameter of the vessel enhancement and the thresholding parameters. Changing *σ* can result in 5% change in sensitivity, while changing most of the other parameters resulted in a small variation in both sensitivity and specificity with an accuracy change under 0.1%.

Based on the results of Table 8, the pre- and postprocessing steps applied in the proposed method increased the overall accuracy of the segmentation by 1% to 2% by removing unwanted objects, filling some holes caused by specular reflexes, and smoothing the vessel edges.

## 9. Conclusion

In this paper we presented a multiresolution method for segmenting blood vessels in fundus photographs. The proposed method and the Frangi method were evaluated using multiple online available databases with diverging image resolution. The proposed algorithm shows in each case an increase both in sensitivity and accuracy to segment vessels compared to the Frangi method with a decreased computational complexity.

This gain in accuracy is mainly due to easier handling of central reflexes of thick vessels in lower resolution images, while the computational needs are significantly reduced by using the resolution hierarchy. This can be further improved by parallelization and implementation using a GPU.

With the proposed modifications the algorithm is more applicable in complex automatic systems, and the segmentation results can be used as a basis for other algorithms to analyze abnormalities of the human eye. Additionally we introduced a new high resolution fundus image database [33] to evaluate segmentation and localization methods, where our algorithm reached an accuracy of over 96% on average.

## Figures and Tables

**Table 1:** Details of used databases.

Database	Images used	Resolution
DRIVE [26]	20	565 × 584
STARE [32]	20	700 × 605
High resolution fundus [33]	45	3504 × 2336

**Table 2:** Comparison of the results using the DRIVE [26] public database. The proposed methods achieved the best accuracy (Acc) compared to the state-of-the-art solutions.

Algorithm	Se	Sp	Acc
Proposed	0.644	**0.987**	**0.9572**
Frangi et al. [1]	0.660	0.985	0.9570
Marín et al. [34]	0.706	0.980	0.945
Human observer	0.776	0.972	0.947
Dizdaroglu et al. [24]	0.718	0.974	0.941
Soares et al. [35]	0.7283	0.9788	0.9466
Mendonça and Campilho [36]	**0.7344**	0.9764	0.9452
Staal et al. [37]	0.7194	0.9773	0.9442
Niemeijer et al. [38]	—	—	0.9416
Zana and Klein [39]	—	—	0.9377
Martinez-Perez et al. [40]	0.7246	0.9655	0.9344
Odstr$\overset{ˇ}{\text{c}}$ilík et al. [7]	0.7060	0.9693	0.9340
Espona et al. (subpixel accuracy) [41]	0.7313	0.9600	0.9325
Chaudhuri et al. [4]	0.6168	0.9741	0.9284
Al-Diri and Hunter [22]	—	—	0.9258
Espona et al. (pixel accuracy) [41]	0.6615	0.9575	0.9223
Jiang and Mojon [42]	—	—	0.9212
All background	—	—	0.8727

“—” indicates that this information was not available.

**Table 3:** Sensitivity (Se), specificity (Sp), and accuracy (Acc) of the methods measured on the STARE [32] database. The proposed modifications improved both the sensitivity and accuracy of the Frangi method.

Algorithm	Se	Sp	Acc
Proposed	0.58	0.982	0.9386
Frangi et al. [1]	0.529	**0.986**	0.9370
Marín et al. [34]	0.694	0.981	0.952
Staal et al. [37]	0.6970	0.9810	**0.9516**
Zhang et al. [12]	0.07177	0.9753	0.9484
Soares et al. [35]	0.7165	0.9748	0.9480
Mendonça and Campilho [36]	0.6996	0.9730	0.9440
Martinez-Perez et al. [40]	0.7506	0.9569	0.9410
Chaudhuri et al. [4]	0.6134	0.9755	0.9384
Human observer	0.8949	0.9390	0.9354
Odstr$\overset{ˇ}{\text{c}}$ilík et al. [7]	**0.7947**	0.9512	0.9341
Hoover et al. [9]	0.6751	0.9367	0.9267

**Table 4:** Overall sensitivity (Se), specificity (Sp), and accuracy (Acc) measured using our high resolution fundus database.

Algorithm	Se	Sp	Acc
Proposed	**0.669**	**0.985**	**0.961**
Frangi et al. [1]	0.622	0.982	0.954
Odstr$\overset{ˇ}{\text{c}}$ilík et al. [7]	0.774	0.966	0.949

**Table 5:** Sensitivity (Se), specificity (Sp), and accuracy (Acc) measured for the three datasets separately in our high resolution database.

Dataset	Algorithm	Se	Sp	Acc
Healthy	Proposed	0.662	**0.992**	**0.961**
Healthy	Frangi et al. [1]	0.621	0.989	0.955
Healthy	Odstr$\overset{ˇ}{\text{c}}$ilík et al. [7]	**0.786**	0.9750	0.953
Glaucomatous	Proposed	0.687	**0.986**	**0.965**
Glaucomatous	Frangi et al. [1]	0.654	0.984	0.961
Glaucomatous	Odstr$\overset{ˇ}{\text{c}}$ilík et al. [7]	**0.790**	0.964	0.949
Diabetic retinopathy	Proposed	0.658	**0.977**	**0.955**
Diabetic retinopathy	Frangi et al. [1]	0.590	0.972	0.946
Diabetic retinopathy	Odstr$\overset{ˇ}{\text{c}}$ilík et al. [7]	**0.746**	0.961	0.944

**Table 6:** Comparison of average runtime using two public databases. The effects of the proposed modifications on the calculation time in seconds as shown.

Algorithm	Runtime (in sec)		Accuracy	
	STARE	DRIVE	STARE	DRIVE
Frangi et al. [1]	1.62	1.27	0.9370	0.9570
Proposed	1.31	1.04	0.9386	0.9572
Espona et al. (subpixel accuracy)* [41]	—	31.7	—	0.9325
Mendonça and Campilho* [36]	—	150.0 [35]	0.9480	0.9466
Soares et al.* [35]	180.0	180.0	0.9480	0.9466
Staal et al.* [37]	—	900.0 [35]	0.9516	0.9442

Entries marked by “*” are results reported in the cited articles.

**Table 7:** Comparison of average runtime using high resolution (3504 × 2336) images.

Algorithm	Average runtime	Accuracy
Proposed method	26.693 ± 0.92 sec	0.961 ± 0.006
Original Frangi	39.288 ± 2.00 sec	0.954 ± 0.008
Odstr$\overset{ˇ}{\text{c}}$ilík et al. [7, 33]	18 minutes	0.949

**Table 8:** Accuracy comparison of different settings using the high resolution fundus database.

Algorithm	Accuracy	Absolute change
Proposed method	0.9618 ± 0.0065	—
Without preprocessing	0.9558 ± 0.0064	0.62%
Thresholds decreased by 1%	0.9614 ± 0.0061	0.04%
Thresholds increased by 1%	0.9607 ± 0.0062	0.11%
Without postprocessing	0.9401 ± 0.0085	2.25%
Doubled morphology kernel size	0.9616 ± 0.0060	0.02%
*σ* = 2.0 for Hessian	0.9621 ± 0.0062	0.03%
*σ* = 3.0 for Hessian	0.9621 ± 0.0061	0.03%
*σ* = 4.0 for Hessian	0.9617 ± 0.0064	0.01%
